# Plastic Anisotropy Effect on Variation of Mechanical and Structural Properties of VT23 Titanium Alloy Subjected to Impact-Oscillatory Loading

**DOI:** 10.3390/ma15165718

**Published:** 2022-08-19

**Authors:** Mykola Chausov, Andrii Pylypenko, Pavlo Maruschak, Janette Brezinová, Jakub Brezina, Ihor Konovalenko

**Affiliations:** 1 Department of Mechanics, National University of Life and Environmental Sciences of Ukraine, Heroiv Oborony Str. 15, 03041 Kyiv, Ukraine; 2 Department of Industrial Automation, Ternopil National Ivan Puluj Technical University, Rus’ka Str. 56, 46001 Ternopil, Ukraine; 3 Department of Technology, Materials and Computer Supported Production, Faculty of Mechanical Engineering, Technical University of Košice, Mäsiarska 74, 04001 Košice, Slovakia

**Keywords:** titanium alloy, dynamic non-equilibrium process, mechanical properties, fracture

## Abstract

The main regularities in the impact of varying intensity impact-oscillatory loading on the variation of the mechanical and structural properties of the VT23 high-strength two-phase transverse-rolled sheet titanium alloy have been found. The intensity of the impulse introduction of energy into the alloy under the dynamic non-equilibrium process (DNP) was estimated by ε_imp_ (the increment of dynamic strain). The pulse intensity was found to change the shape of the static strain diagram with further tensioning, as compared to the initial state. This indicates the effect from the structure self-organization inherent in the VT23 titanium alloy upon the DNP. After the DNP (ε_imp_ = 1.44%), with further static deformation, the tensile diagram revealed yield sites up to 6.5% long. In most cases, the DNP was found to have a negative effect on the variation of the mechanical properties of the VT23 titanium alloy, especially if the latter was rolled in the transverse direction. The optimal DNP intensity is ε_imp_~1.5%. In this case, the DNP can be used as an effective plasticization technology for the VT23 titanium alloy (regardless of the rolling direction) in the stamping of high-strength titanium alloys. Changes in the mechanical and structural condition of the VT23 titanium alloy subjected to the DNP were confirmed by the fractographic investigation of specimen fractures.

## 1. Introduction

Titanium alloys are widely used for various-purpose air transport and aerospace systems, special equipment, and deep-sea submarines [1,2,3]. Their high strength, corrosion resistance, and wide temperature range make it possible to significantly reduce the structure’s weight and enhance the reliability of its operation.

Products made of titanium alloy can be subjected to dynamic loads over a wide range of strain rates. Therefore, many works are dedicated to changes in the mechanical properties of titanium alloys depending on the load rate and temperature [4,5,6,7,8,9,10,11,12,13,14,15,16,17,18,19,20,21,22,23,24,25,26,27]. Notably, the ability to self-organize inherent in the structure of high-strength titanium alloys upon the DNP has not been given proper attention so far [28]. The DNP is associated with the fast energy exchange between the system and the environment, and some features of the mechanical system’s parameters, mainly their natural frequency. These differences (features) are mostly transient; they appear instantly due to a particular initial condition and vanish when the balance between the system and the environment is attained. The term “self-organization” has been generally accepted since the middle of the 20th century. It was introduced by the works of Haken, Ebeling, Nikolis and Prigogine to denote the main characteristic of a well-ordered structure formed in conditions of thermodynamic systems far from equilibrium. In Solid State Physics, it refers to the possibility of a well-ordered defect formed in a structure to promote the hydrodynamic plastic flow that reduces the dislocation slide. More specifically, it characterizes the hydrodynamic plastic flow rather than the dislocation slide [29]. Interestingly, such processes can be realized in materials of any statically indeterminate mechanical system when additional force energy is introduced into this system in a pulsed manner, for instance, due to the application of additional impulse loads. Given its great practical and scientific value, testing materials under the DNP requires further research.

Chausov et al. [30,31,32] propose the DNP realization method using impulse force loads under impact-oscillatory loading and tested it on materials of different classes. The basis for the method’s development was the fact that strain rates of various materials, at which self-organizing processes are taking place, with new spatial dissipative structures being formed under impact-oscillatory loading, have their ranges. In particular, the Phantom v711 high-speed camera (maximum registration speed: 1,400,000 k/s) helped reveal two stages of the low- and high-speed processes responsible for the formation and development of a dissipative structure in materials under impact-oscillatory loading. The mean strain rate at the first stage of the process is 1–3 s^−1^ for aluminum alloys D16 and 2024-T3 [32] and 1–2 s^−1^ for stainless steel 12Kh17, respectively [33]. In the second stage, where the newly formed dissipative structure is likely to spread across the specimen’s volume, a sharp increase in the strain rate is observed in local areas up to 50–60 s^−1^ for aluminum alloys D16 and 2024-T3 [32] and up to 6–10 s^−1^ for the 12Kh17 stainless steel [33]. That is, these processes primarily depend on the mechanical and structural properties of the material under study. We should also note that these processes occur at relatively low rates of additional impulse loads, when the mass transfer occurs in the absence of the energy dissipation in the classical sense (conversion of mechanical energy into heat). The oscillating load frequency, which occurs during the DNP of materials according to the proposed method, was found to be in the range of 1–2 kHz [32]. An experiment found that polycrystals have specific intensity ranges for introducing impulse energy into the material, which under further loading can show an increase or decrease in its ductility, toughness and static impact toughness. We should note that these effects may affect the strength of materials. Although most experiments were conducted at room temperature, many studies on titanium and aluminum alloys exposed specimens to temperatures of 77 K prior to the impulse introduction of energy into the specimen under the DNP and after this procedure [34,35]. The mechanical behavior patterns of materials after the DNP observed in the experiment indicate:

- The hazardous nature of additional impulse loads when applied to load-bearing structures during operation, as their mechanical properties can deteriorate significantly;
- The possibility of developing a technology for improving the mechanical properties of the source material using a simple operation of preliminary introduction of impulse energy in a given intensity range.

The authors performed many experiments to evaluate changes in the mechanical and structural parameters of high-strength two-phase titanium sheet alloys VT22, VT23, and VT23M under the DNP. However, most experiments were performed on specimens cut out of titanium sheets rolled in the longitudinal direction [36]. Given that all titanium alloys are anisotropic materials, it is advisable to study the DNP effect on specimens of titanium alloys rolled in the transverse direction.

This research aims to evaluate and generalize the anisotropy effect on the variation of the mechanical and structural properties (α + β) of titanium alloy VT23 after the DNP.

## 2. Materials and Methods

The mechanical testing technique was implemented based on a modified ZD-100Pu (WPM, Leipzig, Germany) hydraulic installation for static testing and is described in detail in [30,31,32]. The proposed technique’s main idea consists of high-speed tensioning of the material with the imposition of a high-frequency (1–2 kHz) oscillatory process, which corresponds to the natural frequency of the testing machine. Structurally, this is achieved by an inner contour introduced into the testing machine in addition to the outer contour (loaded frame of the testing machine). The inner contour is the simplest statically indeterminate structure in the form of three parallel elements loaded simultaneously—the central specimen and two satellites (brittle samples) of different cross-sections made of hardened steels 65 G or U8–U12. When this structure is tensioned, the satellites are destroyed, and the energy is introduced into the specimen under study in a pulsed manner. Satellites may get involved in the operation at any stage of preliminary static tension, making it possible to study the effect of the impulse introduction of energy on the degradation of mechanical properties by the material getting damaged in the process of static tension. By changing the initial diameter of the satellites, it is possible to control the intensity of the impulse introduction of force energy into the material.

**Figure 1:**
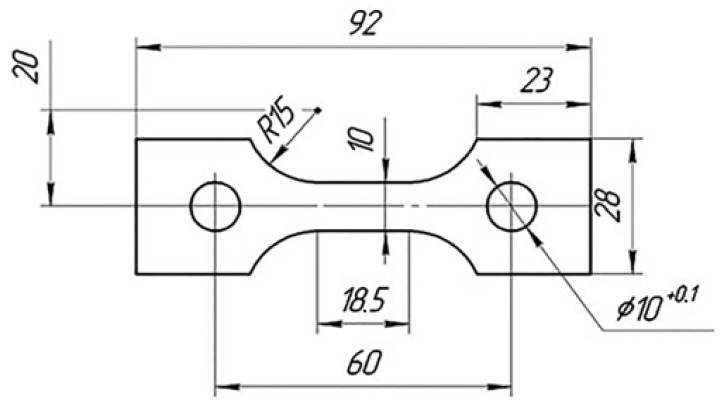
Test specimen (dimensions indicated in mm).

Mechanical tests were performed on specimens (Figure 1) made of the VT23 sheet industrial titanium alloy with a thickness of 3 mm at a room temperature. The strain measurement base was 16 mm. In this case, a standard 0.5 accuracy class extensometer manufactured at the Antonov aircraft production plant, Ukraine, was used for strain measurements.

The value of ε_imp_ under the DNP was controlled by the optical method [32].

The initial mechanical properties of the alloy rolled in different directions are given in Table 1.

The chemical composition of alloy VT23 is given in Table 2.

The content of the α and β phases (in %) in the VT23 titanium alloy in the initial state was evaluated according to the data of radiographic studies [36]. The diffractometric study showed that the β phase occupies 43% of the mass in the VT23 titanium alloy, and the α phase occupies 57%. Characteristically, both phase components have a texture in the crystallographic direction of the specimen (002). This may be due to rolling or subjecting the specimens to another mechanical impact.

Fractographic studies of specimen fractures to detect morphological differences in the fracture micromechanisms were performed on the Zeiss EVO-40XVp (ZEISS International, Oberkochen, Germany) scanning electron microscope. An automated method for analyzing the shape and size of dimples of ductile tearing formed during static and impact fracture of titanium alloys VT23 was used. The method is based on the analysis of the image topology. The method contains the operations of smoothing the initial fractographic image; its convolution with a filter to identify topological ridges; thresholding with subsequent skeletonization to identify boundaries between dimples; clustering to isolate the connected areas that represent the sought objects—dimples. For each dimple, the following quantitative characteristics were calculated: dimple equivalent diameter *deq* and shape parameter *Kc*.

To determine to what degree the shape of dimples approaches the circle, calculated the coefficient of roundness for each *i*-th object. $K_{ci}$ is the percentage of object pixels that fall in a circle with the same area, whose center is combined with the center of mass of the object [37]:

(1) $$K_{ci}=\frac{\sum_{m=1}^{f_{i}}g\left(\vec{r_{m}},d_{i}\right)}{f_{i}}\cdot 100\%,\;g\left(\vec{r_{m}},d_{i}\right)=\{\begin{array}{c} 1,\;при\;\left|\vec{r_{m}}\right|\leq d_{i}/2\\ 0,\;при\;\left|\vec{r_{m}}\right|>d_{i}/2 \end{array}$$

where $f_{i}$ is the number of object pixels; $d_{i}$ is the diameter of the equivalent circle; $g\left(\vec{r_{m}},d_{i}\right)$ is the indicator function that shows whether the *m*-th pixel of the object falls within the boundary of the equivalent circle; $\vec{r_{m}}$ is the radius vector directed from the center of the equivalent circle $C_{i}\left(x_{ci},y_{ci}\right)$ to the *m*-th pixel of the object with coordinates $\left(x_{cm},y_{cm}\right)$ [38,39].

## 3. Results

### 3.1. Mechanical Properties

**Figure 2:**
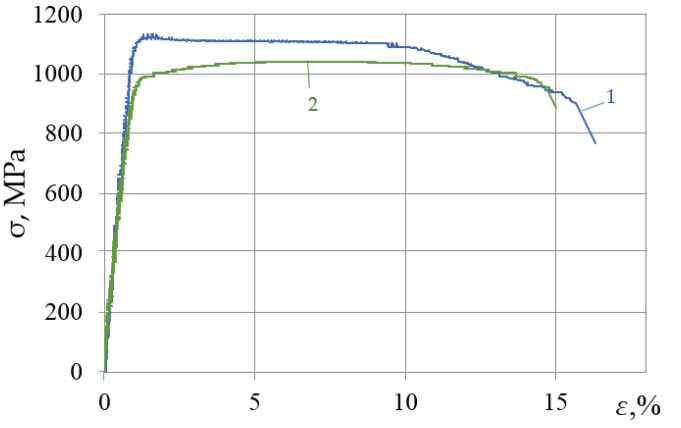
Static tensioning of specimens: 1—longitudinal rolling; 2—transverse rolling.

Figure 2 shows stress–strain diagrams of specimens from the VT23 titanium alloy loaded by static tensioning. The specimens are cut out of the sheet material and rolled in different directions. The effect of structural anisotropy on mechanical properties is revealed. It is noteworthy that in the longitudinal direction of rolling, the stress–strain diagram has almost no hardening area of the alloy. Immediately after attaining the tensile strength, a yield site occurs, after which the material is strengthened. In the transverse direction of rolling, the VT23 titanium alloy has a lower strength, but the diagram has a fairly long hardening area of the alloy, which indicates its strong load-bearing capacity.

The alloy’s total plasticity in different rolling directions is relatively uniform. Given these differences in the mechanical properties of the specimens rolled in different directions, we should expect significant differences in the variation of the mechanical and structural parameters after the DNP. For a detailed analysis of the DNP effect, the dynamic strain increments (ε_imp_) of specimens in the process of impulse introduction of energy were set as (ε_imp_ = 0.8–5.5%). Given that at ε = 5.5%, the material’s ultimate strength is nearly attained on the static tension diagram of the specimens, the maximum ε_imp_ was chosen. Previous DNP studies have shown that at such high stresses, the repeated static tensioning of titanium alloys has revealed deterioration of their mechanical properties.

**Figure 3:**
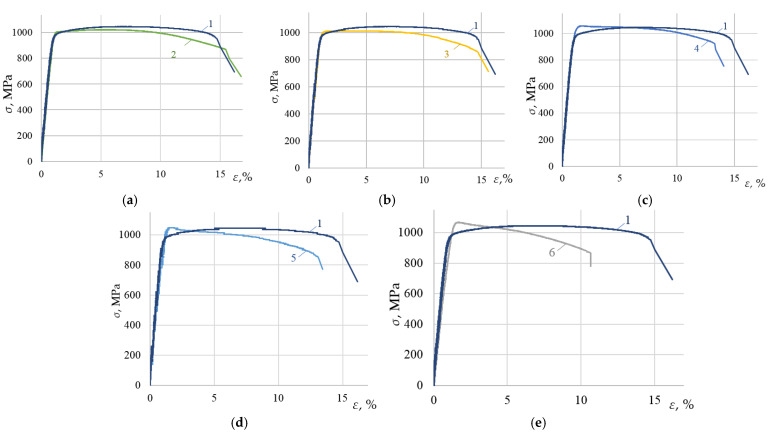
Stress–strain diagrams of the VT23 titanium alloy rolled in the transverse direction after the DNP and subsequent static tensioning, as compared to the static tensioning in the initial state (curve 1): (**a**) (2—ε_imp_ = 0.81%); (**b**) (3—ε_imp_ = 1.44%); (**c**) (4—ε_imp_ *=* 2.56%); (**d**) (5—ε_imp_ *=* 4.56%); (**e**) (6—ε_imp_ = 5.5%).

Figure 3a–e give examples of the stress–strain diagrams obtained for specimens of the VT23 alloy rolled in the transverse direction after the DNP and repeated static tension. For comparison, all the graphs show a stress–strain diagram of the alloy in the initial state.

**Figure 4:**
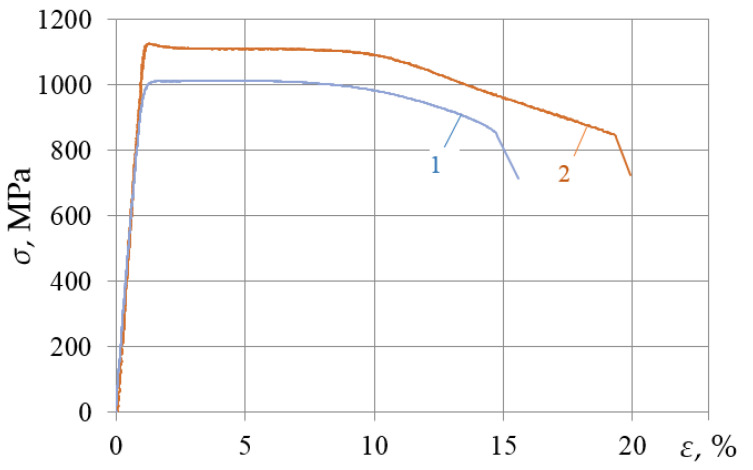
Stress–strain diagrams of the VT23 alloy after the DNP: 1—transverse direction of rolling, ε_imp_ = 1.44%; 2—longitudinal direction of rolling, ε_imp_ = 1.5% [36].

The analysis of the results shows that at ε_imp_ > 2.56%, the mechanical properties of the VT23 titanium alloy deteriorated significantly with repeated static tensioning. Most interesting effects were found at ε_imp_ = 1.44% when yield sites up to 6.5% long were formed on the stress–strain diagram with further static tensioning. At the same time, the yield strength of the alloy increased to 1000 MPa, that is, by 5–6%, compared to the initial state. The ultimate strength of the alloy decreased by approximately the same percentage, and the total ductility remained unchanged. The stress–strain diagram of the VT23 titanium alloy (rolled in the longitudinal and transverse directions) under the DNP (ε_imp_ = 1.44%) and subsequent static tension were compared Figure 4.

The research findings indicate that after the DNP of the same intensity, one can obtain stress–strain diagrams similar in shape for specimens of the VT23 titanium alloy cut in different directions of rolling. At the same time, they both have significant yield areas. This effect can be used for technological purposes.

### 3.2. Fractographic Research Results

**Figure 5:**
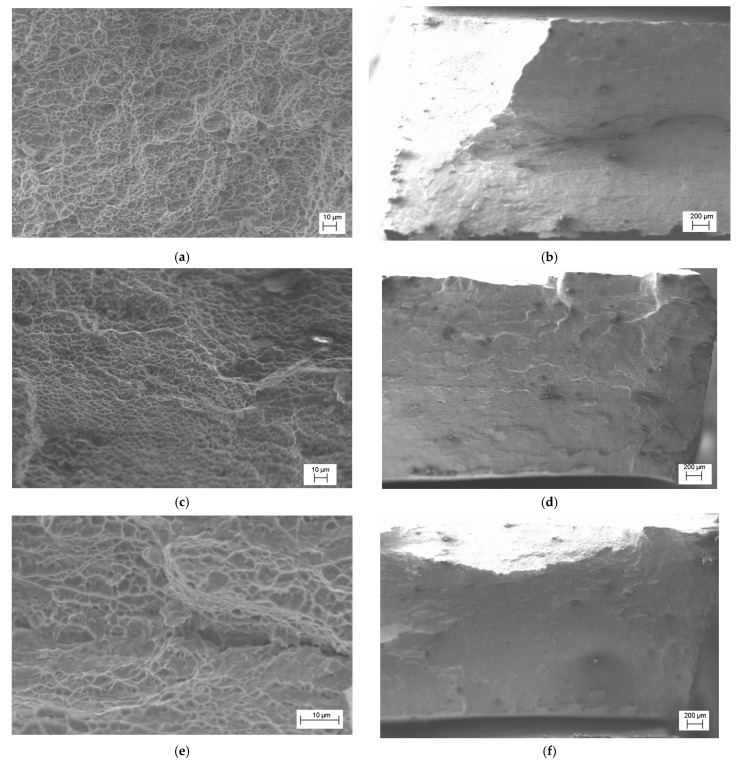
Fracture micromechanisms of specimens from titanium alloy VT23 rolled in the transverse direction after the DNP and subsequent static tensioning: (**a**,**b**)—ε_imp_ = 0.81%; (**c**,**d**)—ε_imp_ = 2.56%; (**e**,**f**)—ε_imp_ = 4.56%.

Characteristic changes in the mechanical properties of the VT23 titanium alloy at different DNP intensities (see Figure 4) are directly related to the characteristic structural changes, as evidenced by the results of fractographic research (Figure 5).

The research was carried out both in the central zones of the specimens, where the fracture occurred by the dimple separation mechanism, and in the lateral areas characterized by shear fracture mechanisms. Figure 5 presents their images. The DNP changed the fracture micromechanisms of the VT23 titanium alloy under study. Thus, at low ε_imp_, static tensioning diagrams are characterized by a slight strengthening of the alloy on the ascending branch of the stress–strain diagram (see Figure 3a). This, in turn, causes an increase in the fibrous cup zone characterized by a significant number of small dimples (Figure 5a,b) in the central zone of the specimen separation. At the same time, apart from viscous chipping areas that occur in different directions, many dimples of different sizes formed on the lateral walls indicate a significant ductility of the alloy’s fracture.

An increase in ε_imp_ (see Figure 5c–f) causes the formation of local micro separation areas in the central zones of the specimen separation. The relief formed by dimples of different sizes changes appreciably, as evidenced by the depth of separation dimples. All this indicates that the accumulated defects that merge into pores and microcracks dominate the descending branches of the stress–strain diagrams, which primarily affects their length (see Figure 3c,d). The flat chipping areas on the lateral walls of the specimens indicate a decrease in the fracture toughness of the alloy with an increase in the DNP intensity.

**Figure 6:**
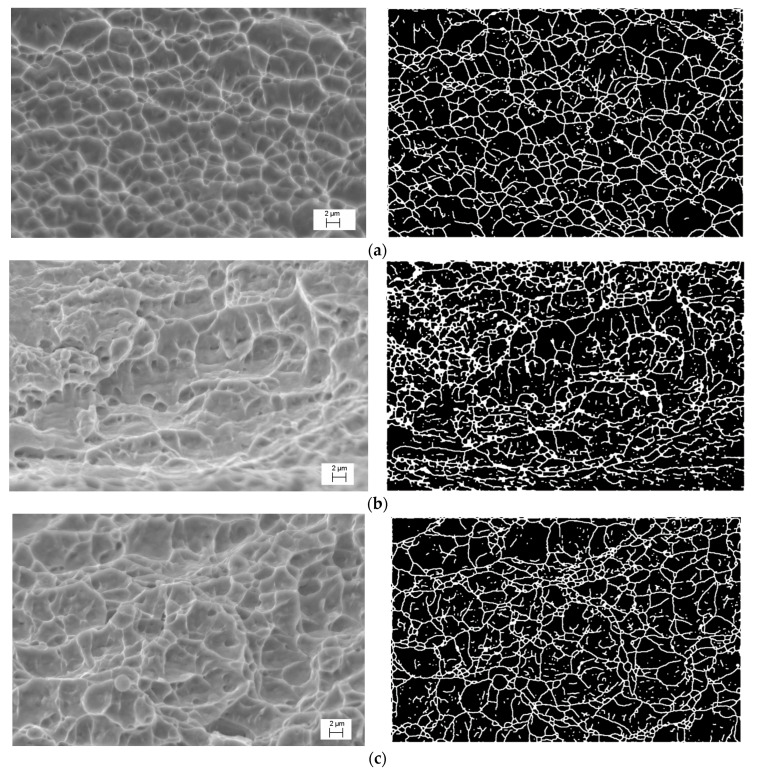
Fractograms of fracture surfaces of specimens made of the VT23 alloy rolled in the transverse direction, and their binarized mappings obtained after the impulse energy introduction under impact-oscillatory loading followed by static tensioning: (**a**)—ε_imp_ = 0.81%; (**b**)—ε_imp_ = 2.56%; (**c**)—ε_imp_ = 4.56%.

Above is the qualitative analysis of the fractographic research findings. For their quantitative analysis, the authors used the designated, highly efficient computer diagnostic system for the automated processing of the images obtained [37,40]. Figure 6 shows the fractographic images of the fracture microrelief formed on specimen surfaces, and their binarized mappings.

**Figure 7:**
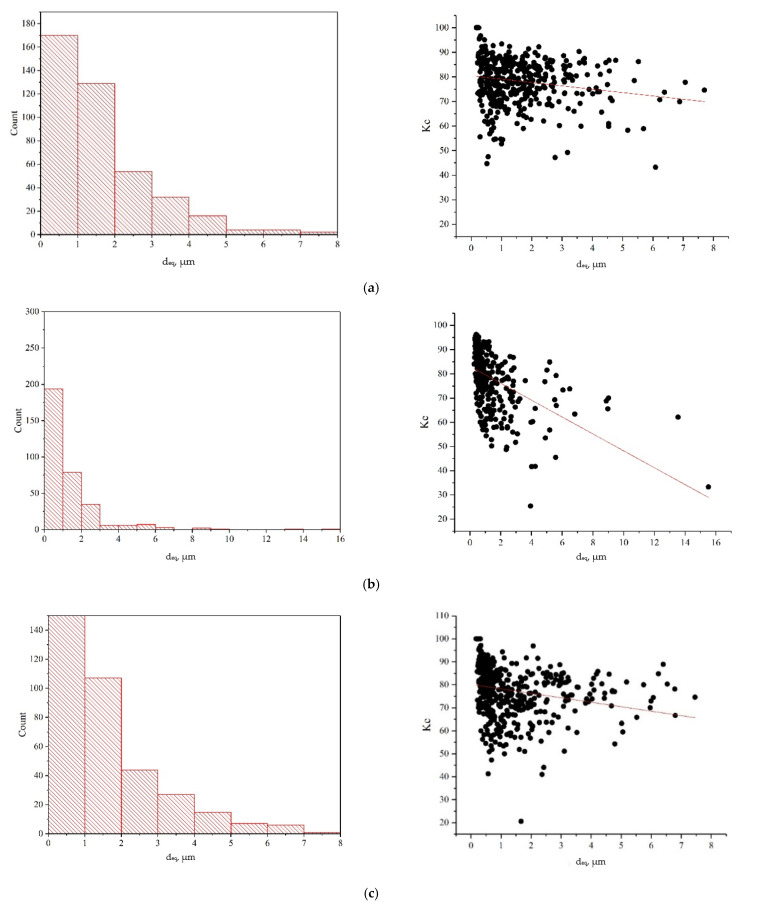
Histograms of dimple diameters and diagrams of dimple shape coefficients on fracture zones of specimens made of the VT23 alloy rolled in the transverse direction after the introduction of impulse energy due to impact-oscillatory loading followed by static tensioning: (**a**)—ε_imp_ = 0.81%; (**b**)—ε_imp_ = 2.56%; (**c**)—ε_imp_ = 4.56%.

It is noteworthy that during the DNP followed by static tensioning, the central fracture zones of specimens made of the VT23 alloy are characterized by dimple microrelief under all loading conditions considered; however, its morphology is somewhat different due to the varying intensity of the impulse energy introduction. In particular, at ε_imp_ = 0.81%, 412 dimples were found within the area analyzed, the diameters of which were mostly up to 5 μm, with the maximum size reaching 8 μm. Of particular note is the shape coefficient parameter, the value of which (Kc = 70–80, according to the approximation) indicates the homogeneity of the dimple geometry and its closeness to the circular shape (see Figure 7a). These data indicate a relatively high ductility and crack resistance of the alloy in this structural state.

The fracture of specimens after the realization of the DNP (ε_imp_ = 2.56%) is also characterized by a dimple structure, which, however, differs significantly from the previous one. The dimples found on the surface do not have a clearly defined shape, as evidenced by the value of the shape coefficient (Kc = 25–85, according to the approximation). A significant scatter of size is inherent in the dimples, while their diameter may reach 16 μm. In addition, zones of elongation [41] were found around the dimples, indicating the alloy strain localization. The presence of such elements and a significantly larger area of membranes between the dimples indicate a decreased microplastic deformation and increased brittle component in the specimen fracture.

Fracture of specimens after the DNP (ε_imp_ = 4.56%) results from the combined effect of the DNP and relaxation processes in the alloy in case of a significant intensity of introduction of impulse energy. On the one hand, the DNP activates deformation processes and fractions (fragments) the original β phase of the alloy, as shown earlier [42]. On the other hand, with a significant intensity of introduction of impulse energy into the alloy, relaxation processes occur in the alloy. Apart from the smaller structural elements formed (grains, subgrains, fragments), these relaxation processes are associated with the activation of rather intense dislocation redistributions, the consequence of which is the annihilation of intra-volume dislocations [42]. This caused the formation of the fracture surface similar to that shown in Figure 6a (the dimple diameter was mostly up to 5 μm, with the maximum size reaching 8 mm), however, with a different shape coefficient of the dimples (Kc = 65–80, according to the approximation). In addition, the number of dimples with sizes larger than 5 μm is different, and one can see that all the dimples have a flat bottom (the dimple diameter is much larger than its depth, compare Figure 6a,c). This indicates the presence of a brittle component in the micromechanism of fracture. Sharp boundaries between the dimples, which are clearly visible on the binarized image, also testify to an appreciable embrittlement of the material (see Figure 6c). Thus, fractographic research indirectly indicates significant structural changes in the alloy caused by the previous impulse introduction of energy of different intensities into the VT23 titanium alloy, which is associated with the self-organization of the alloy structure during the DNP. It should be noted that a similar procedure for evaluating the differences in the fracture relief of specimens made of the VT23 titanium alloy rolled in the longitudinal direction after the DNP of varying intensity was previously described in [43].

## 4. Discussion

**Figure 8:**
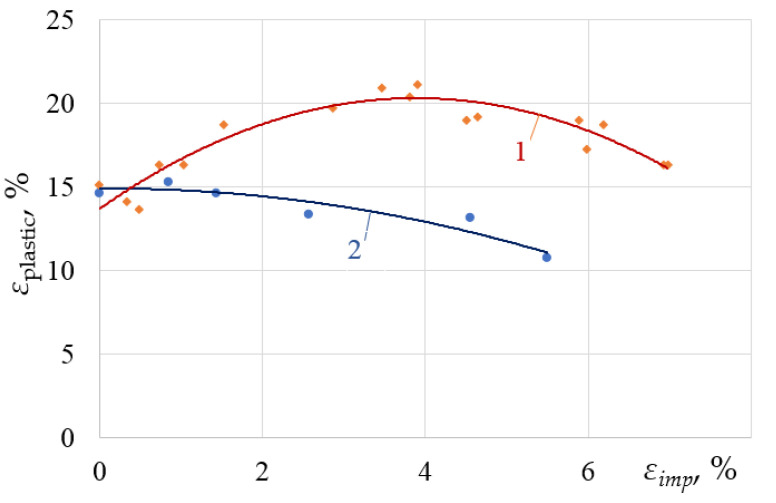
Dependence of the maximum strain of the VT23 alloy rolled in different directions on the increase in the dynamic strain increment ε_imp_ of specimens during the DNP: 1—longitudinal direction. [36]; 2—transverse direction.

To evaluate the effect of anisotropy on the variation of the mechanical properties of the alloy under the DNP, the authors used the previously obtained results for the VT23 titanium alloy rolled in the longitudinal direction [36] (Figure 8) and the test data obtained for specimens cut in the transverse rolling direction described in this article.

Here, the abscissa shows the intensity of impulse energy introduced into the alloy, and the ordinate shows the maximum total deformation of the alloy after repeated static tension.

*The transverse direction of rolling.* In most cases, the previous DNP deteriorated the mechanical properties of the VT23 titanium alloy obtained by subsequent static tension. In addition, specimens cut in the transverse direction of rolling are more sensitive to this phenomenon than those cut in the longitudinal direction [44,45,46,47]. At the same time, a narrow ε_imp_ range was found, in which the ductility of the VT23 titanium alloy can be improved. This range is provided at ε_imp_~1.5% (see Figure 4), when yield areas of significant length appear on the static stress–strain diagrams. An increase in ε_imp_ causes a deterioration in ductility and strength (see Figure 3).

*The longitudinal direction of rolling.* The maximum plastic properties were achieved at ε_imp_ = 4% while maintaining the strength properties for the specimens cut in the longitudinal direction of rolling. With DNP intensity ε_imp_~1.5%, the total deformation of the VT23 titanium alloy increased by 18% (see Figure 2 and Figure 4) due to the yield areas formed, while the strength properties also remained unchanged.

In the authors’ opinion, the range of the impulse introduction of energy (ε_imp_~1.5%) described is optimal in terms of improving the initial mechanical properties of the VT23 titanium alloy rolled in different directions, and can be used as a basis for developing the alloy plasticization technology [48,49].

## 5. Conclusions

The main regularities in the plastic anisotropy effect on the variation of the mechanical and structural properties of the VT23 high-strength two-phase titanium sheet alloy subjected to the DNP are established. For specimens of the VT23 titanium alloy rolled in the transverse direction, the DNP was found to be more harmful than for those rolled in the longitudinal direction. The strength and plastic properties of the VT23 titanium alloy deteriorated over nearly the entire DNP range (ε_imp_ = 0.8–5.5%) investigated. However, at ε_imp_~1.5%, the positive effects of yield areas (up to 6.5% long) were revealed, practically without reducing the total plasticity of the source material. Notably, the impulse introduction of energy into the alloy rolled in the longitudinal direction led to the improved plastic properties of the alloy in a wide range of the ε_imp_ parameter variation (ε_imp_ = 1–7%). In particular, at ε_imp_ = 1.5%, the VT23 titanium alloy’s ductility increased by 18% compared to the initial state. Moreover, specimens rolled in the longitudinal direction at a given DNP intensity were also characterized by yield areas up to 8% long.

Therefore, the optimal DNP range for the VT23 titanium alloy (ε_imp_~1.5%) was found, in which the initial mechanical properties of the alloy rolled in different directions are improved simultaneously, making it possible to use this result for technological purposes. In particular, for developing the stamping technology for high-strength titanium alloys.

## Figures and Tables

**Table 1:** The mechanical properties of titanium alloy VT23 depend on the rolling direction.

Rolling Direction	Mechanical Properties		
	*σ_ys_*, MPa	*σ_us_*, MPa	δ, %
longitudinal	1060–1080	1120–1180	16
transverse	960–980	1040–1080	15

**Table 2:** Chemical composition of alloy VT23.

Fe	Cr	Mo	V	Ti	Al
0.5–0.8	1.0–1.4	1.8–2.5	4.3–5	86–89.3	4.4–6.3

## Data Availability

The data presented in this study are available upon request from the corresponding author.
